# Safe, selective histopathological examination of gallbladder specimens: a systematic review

**DOI:** 10.1002/bjs.11759

**Published:** 2020-07-08

**Authors:** V. P. Bastiaenen, J. E. Tuijp, S. van Dieren, M. G. Besselink, T. M. van Gulik, L. Koens, P. J. Tanis, W. A. Bemelman

**Affiliations:** ^1^ Department of Surgery, Cancer Centre Amsterdam Amsterdam UMC Amsterdam the Netherlands; ^2^ Department of Pathology, Amsterdam UMC University of Amsterdam Amsterdam the Netherlands

## Abstract

**Background:**

Routine histopathological examination after cholecystectomy is costly, but the prevalence of unsuspected gallbladder cancer (incidental GBC) is low. This study determined whether selective histopathological examination is safe.

**Methods:**

A comprehensive search of PubMed, Embase, Web of Science and the Cochrane Library was performed. Pooled incidences of incidental and truly incidental GBC (GBC detected during histopathological examination without preoperative or intraoperative suspicion) were estimated using a random‐effects model. The clinical consequences of truly incidental GBC were assessed.

**Results:**

Seventy‐three studies (232 155 patients) were included. In low‐incidence countries, the pooled incidence was 0·32 (95 per cent c.i. 0·25 to 0·42) per cent for incidental GBC and 0·18 (0·10 to 0·35) per cent for truly incidental GBC. Subgroup analysis of studies in which surgeons systematically examined the gallbladder revealed a pooled incidence of 0·04 (0·01 to 0·14) per cent. In high‐incidence countries, corresponding pooled incidences were 0·83 (0·58 to 1·18), 0·44 (0·21 to 0·91) and 0·08 (0·02 to 0·39) per cent respectively. Clinical consequences were reported for 176 (39·3 per cent) of 448 patients with truly incidental GBC. Thirty‐three patients (18·8 per cent) underwent secondary surgery. Subgroup analysis showed that at least half of GBC not detected during the surgeon's systematic examination of the gallbladder was early stage (T1a status or below) and of no clinical consequence.

**Conclusion:**

Selective histopathological examination of the gallbladder after initial macroscopic assessment by the surgeon seems safe and could reduce costs.

## Graphical abstract

This meta‐analysis found that the incidence of truly incidental gallbladder cancer (GBC) was less than 0·5 per cent and decreased to less than 0·1 per cent when the surgeon performed a systematic macroscopic examination. At least 50 per cent of GBC that was not detected before or during surgery was of early stage and with no clinical consequences. Particularly in non‐endemic regions, selective histopathological examination after initital macroscopic assessment of the gallbladder by the surgeon seems safe and will likely result in significant cost and time savings.

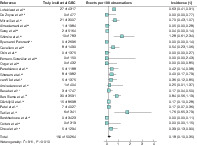

Open and palpate it

## Abstract (es)

**Antecedentes:**

El examen histopatológico rutinario después de la colecistectomía es caro y la prevalencia de cáncer de vesícula biliar (*gallbladder cancer*, GBC) no sospechado o incidental es baja. Este estudio determinó si el examen histológico selectivo es seguro.

**Métodos:**

Se realizó una búsqueda exhaustiva en PubMed, Embase, Web of Science y en la Biblioteca Cochrane. Se estimaron las incidencias agrupadas de GBC incidental y realmente incidental (GBC detectado durante el examen histopatológico sin sospecha previa o intraoperatoria) utilizando un modelo de efectos aleatorios. Además, se evaluaron las consecuencias clínicas del GBC realmente incidental.

**Resultados:**

Se incluyeron 73 estudios (*n* = 232.155). En los países de baja incidencia, la incidencia agrupada fue de 0,32% para el GBC incidental (i.c. del 95% 0,25 a 0,42) y de 0,18% (0,10 a 0,35) para GBC realmente incidental. El análisis de subgrupos que incluye estudios en los que los cirujanos examinaron sistemáticamente la vesícula biliar reveló una incidencia agrupada de 0,04% (0,01 a 0,14). En los países de alta incidencia, las incidencias agrupadas correspondientes fueron 0,83% (0,58 a 1,18), 0,44% (0.2 a 0.91) y 0,08% (0,02 a 0,39), respectivamente. Se describieron consecuencias clínicas en 176 (39,3%) de 448 pacientes con GBC realmente incidental. Treinta y tres pacientes (18,6%) se sometieron a cirugía secundaria. El análisis por subgrupos mostró que al menos la mitad de los GBC no detectados durante el examen sistemático de la vesícula biliar por parte del cirujano eran tumores de estadio precoz (≤ T1a) y sin consecuencias clínicas.

**Conclusión:**

El examen histológico selectivo de vesículas biliares después de la evaluación macroscópica inicial realizada por el cirujano parece seguro y podría reducir los costes.

## Introduction

Cholecystectomy for benign gallbladder disease is among the most frequently performed surgical procedures worldwide. For decades, it has been standard practice to send all gallbladder specimens for histopathological examination in order to exclude the presence of gallbladder cancer (GBC). This routine policy is costly and constitutes a significant workload for pathology departments, whereas the prevalence of unsuspected gallbladder cancer (incidental GBC) is low. To save costs and reduce the workload of pathologists, selective histopathological examination has been proposed^1^, ^2^, ^3^, ^4^, ^5^. This entails macroscopic examination by the surgeon, sending only specimens with suspicious findings to the pathologist. Some fear that surgeons might not recognize GBC, resulting in missed diagnoses with potentially disastrous consequences. However, previous studies^6^, ^7^ have shown that gallbladder specimens without macroscopic abnormalities rarely contain GBC. Moreover, if no macroscopic abnormalities are present, the cancer is usually of early stage and without clinical consequence, as the previously performed cholecystectomy suffices. In 2014, the Dutch guideline for gallstone disease was updated, proposing selective histopathological examination after cholecystectomy^8^, but implementation was suboptimal^9^. Less than half of hospitals adopted the selective strategy, and the need for more evidence on safety was expressed.

## Methods

This systematic review and meta‐analysis adhered to the Meta‐analysis Of Observational Studies in Epidemiology (MOOSE) guidelines^10^ and the PRISMA statement^11^. The protocol of this systematic review was registered in the PROSPERO international prospective register of systematic reviews (registration number: CRD42019142696). Institutional review board approval was not required.

### Search strategy and selection criteria

A systematic literature search of PubMed, Embase, Web of Science and the Cochrane Library was conducted without language restriction and with a time limit set for studies published since 1 January 2009. The search was last updated on 1 June 2019. The search was performed with the assistance of a clinical librarian and included both medical subject heading (MeSH) terms and keywords. The search terms used were gallbladder (MeSH), cholecystitis (MeSH), cholecystectomy (MeSH), pathology (MeSH), gall bladder, histology, histopathology, routine, selective, carcinoma and cancer. Further details of the search can be found in *Appendix* *S1* (supporting information). The search results were imported into Covidence for further selection^12^. Cross‐referencing of all included articles was performed to find any additional studies of interest. Articles published in languages other than English were translated with the help of native speakers.

All observational studies reporting the number of patients with GBC diagnosed during or after cholecystectomy performed for presumed benign gallbladder disease were eligible for inclusion. Studies that did not mention the total number of patients undergoing cholecystectomy, or the number of patients with GBC that was suspected or diagnosed before surgery, were excluded. In addition, studies that included specimens retrieved from cholecystectomy performed as an incidental procedure during other abdominal or pelvic operations were excluded. Preoperative gallbladder imaging was probably not done when the decision to perform a cholecystectomy was made during surgery, and this might have raised suspicion of GBC in these patients. Other exclusion criteria were: conference abstracts, no original data, no full text available, study performed in a centre with a selective policy of histopathological examination, studies that included post‐mortem specimens, and studies including specimens retrieved from cholecystectomies performed as part of more extensive, usually oncological, surgery (such as the Whipple procedure) rather than because of presumed benign gallbladder disease.

Two reviewers independently screened the titles and abstracts of all retrieved references to identify studies that potentially met the inclusion criteria. Subsequently, the full texts of the remaining articles were independently assessed for eligibility by the same two reviewers. During all stages of the selection process, any conflict between the reviewers was resolved by discussion until consensus had been reached.

### Definitions and data extraction

For the purpose of this study, GBC included primary gallbladder malignancies regardless of histological type, gallbladder metastases and lymphoid malignancies. Incidental GBC was defined as GBC diagnosed during cholecystectomy or at histopathological examination, with no preoperative suspicion. Within this group, truly incidental GBC was defined as GBC diagnosed for the first time during histopathological examination without any suspicion before or during operation.

Two reviewers independently extracted data from the included studies according to a predefined data extraction table. In case of disagreement, discussion took place until consensus had been reached. Collected data included study characteristics (authors, publication year, study country, study design, study period and sample size), the number of patients with (truly) incidental GBC and T category, age and sex of patients with truly incidental GBC, the consequences of truly incidental GBC for further management, and authors' recommendations about routine or selective histopathological examination. Study authors were contacted in case of missing data or uncertainty.

### Data analysis

Two reviewers critically appraised all studies according to the Joanna Briggs Institute Critical Appraisal Checklist for Studies Reporting Prevalence Data^13^. This tool consists of nine domains, and the final score for each study ranges from 0 to 9. The risk of bias was rated as high if the final score was 0–4, moderate if the final score was 5–7, and low for a final score of 8 or 9. All studies, regardless of their risk of bias, were included for data analysis.

Because of known variation in the geographical incidence of GBC, included studies were assigned to one the following two groups: studies from countries with a low incidence of GBC, and studies from countries with a high incidence of GBC. Allocation of studies into one of the groups was based on the age‐standardized incidence of GBC, as reported by GLOBOCAN 2018^14^. A cut‐off value of 2·3 per 100 000 was chosen, as this was the age‐standardized incidence of GBC worldwide. As previous epidemiological studies^15^, ^16^, ^17^ had also identified India and Pakistan as countries with a high incidence of GBC, studies from these countries were also assigned to group 2.

The main outcomes were: incidence of incidental GBC; incidence of truly incidental GBC; the ability of the surgeon to recognize GBC during surgery; and the clinical consequences of truly incidental GBC. Additionally, authors' recommendations regarding histopathological examination (routine *versus* selective) and the costs of routine histopathological examination to achieve clinical benefit in one patient were evaluated.

To establish the incidence of incidental GBC, all studies reporting the number of patients with GBC that was not suspected or diagnosed before surgery were included. Results of preoperative imaging that could have raised suspicion (such as a thickened gallbladder wall) were not reinterpreted by the authors as preoperative suspicion, when it was explicitly mentioned that GBC was not suspected before the operation. Studies that excluded patients with GBC suspected or diagnosed during surgery were not included in this analysis.

To establish the incidence of truly incidental GBC, all studies reporting the number of patients with GBC that was not suspected or diagnosed before or during surgery were included. Surgical procedures that required conversion (for example due to dense adhesions) were not reinterpreted as intraoperative suspicious findings, if it was not explicitly mentioned in the article that the surgeon suspected GBC during surgery. Likewise, macroscopic abnormalities reported by the pathologist were not considered as intraoperative suspicious findings if the article did not explicitly mention that these were also detected by the surgeon and had raised suspicion during the operation.

To assess the ability of the surgeon to recognize GBC during surgery, all studies reporting both the number of GBCs suspected or diagnosed during cholecystectomy (with or without opening the specimen) and the number of GBCs diagnosed during histopathological examination were included. Intraoperative sensitivity was defined as the fraction of incidentally detected cancers that were suspected by the surgeon during surgery.

The clinical consequences of truly incidental GBC were grouped into seven categories: no further treatment; periodic surveillance; referral to oncologist (chemotherapy); referral to oncologist (unknown outcome); secondary surgery without 1‐year survival; secondary surgery with unknown survival (survival not reported or follow‐up of less than 1 year); and secondary surgery with at least 1‐year survival. In some studies, all patients were referred to the oncologist to discuss further management, regardless of tumour stage. When the oncologist decided that no additional diagnostic tests or any treatment were indicated, patients were assigned to the first group (no further treatment). Patients who received adjuvant or palliative chemotherapy were appointed to the third group (chemotherapy). If it was unclear whether the referral to the oncologist had led to additional diagnostic tests or treatment, patients were assigned to the fourth group (unknown outcome).

To determine the value of systematic macroscopic examination by the surgeon, subgroup analyses were performed including the studies that specifically described that surgeons opened and systematically examined the mucosa of all removed gallbladders.

To estimate the costs of routine histopathological examination to achieve clinical benefit in one patient, all studies that reported both the number of specimens with macroscopic abnormalities detected by the surgeon during systematic assessment of the gallbladder and the number of patients with truly incidental GBC were included. The analysis was based on the assumptions that: in the Netherlands, costs for histopathological examination are approximately €60 per gallbladder specimen; in a hypothetical situation of a selective policy, only specimens with macroscopic abnormalities are sent for histopathological examination; in the case of routine policy, all gallbladder specimens are sent for histopathological examination; and clinical benefit can be achieved only in patients with GBC of T1b status or above, as no further treatment is indicated for patients with less advanced GBC. To estimate the costs of a routine policy to achieve clinical benefit in one patient, the difference between costs made with a routine and selective policy was calculated and then divided by the number of patients with a normal‐looking gallbladder containing GBC of at least T1b category (GBC with clinical consequences that would have been missed with a selective policy).

### Statistical analysis

The incidence of (truly) incidental GBC and the intraoperative sensitivity were meta‐analysed. Pooled percentages with corresponding 95 per cent c.i. were calculated using a generalized linear mixed model. A random‐effects model was used, considering the expected variation between the studies. Heterogeneity of the included studies was evaluated by calculating the *I* ^2^ index and corresponding *P* value. All statistical analyses were performed using RStudio version 1.1.453 (R Core Team, 2018; R Foundation for Statistical Computing, Vienna, Austria). The numbers of patients per category of clinical consequences of truly incidental GBC were reported as absolute numbers and percentages.

## Results

**Fig. 1:**
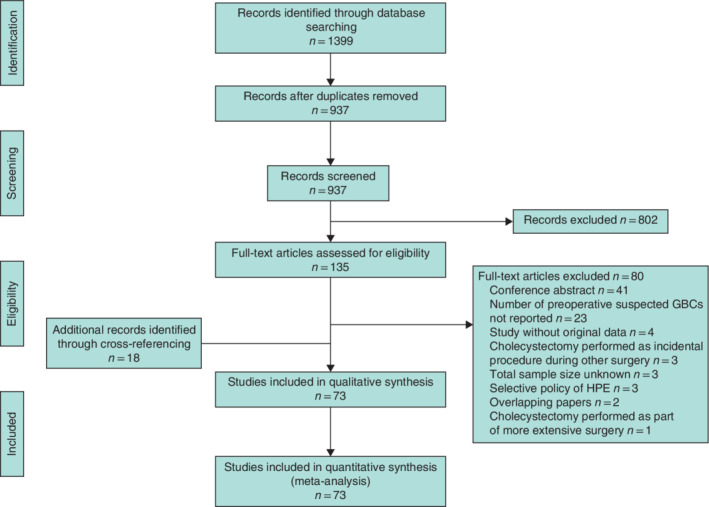
PRISMA diagram for the review GBC, gallbladder cancer; HPE, histopathological examination.

In total, 937 studies were identified after removal of duplicates. Based on title and abstract, 135 studies were selected for full‐text assessment. Eighty articles were excluded because they did not meet the eligibility criteria (*Fig*. *1*). After full‐text screening, 55 articles met the eligibility criteria for this systematic review. An additional 18 studies were identified during manual cross‐referencing, resulting in a total of 73 articles, including 232 155 patients, that were eligible for data analysis. These studies were then divided into those performed in countries with a low incidence (39)^6^, ^18^, ^19^, ^20^, ^21^, ^22^, ^23^, ^24^, ^25^, ^26^, ^27^, ^28^, ^29^, ^30^, ^31^, ^32^, ^33^, ^34^, ^35^, ^36^, ^37^, ^38^, ^39^, ^40^, ^41^, ^42^, ^43^, ^44^, ^45^, ^46^, ^47^, ^48^, ^49^, ^50^, ^51^, ^52^, ^53^, ^54^, ^55^ and countries with a high incidence (34)^7^, ^56^, ^57^, ^58^, ^59^, ^60^, ^61^, ^62^, ^63^, ^64^, ^65^, ^66^, ^67^, ^68^, ^69^, ^70^, ^71^, ^72^, ^73^, ^74^, ^75^, ^76^, ^77^, ^78^, ^79^, ^80^, ^81^, ^82^, ^83^, ^84^, ^85^, ^86^, ^87^, ^88^ of GBC. Characteristics of all included studies are presented in *Table S1* (supporting information). Critical appraisal showed that four studies were judged to have a high risk of bias, 51 were classified as having a moderate risk of bias, and 18 had a low risk of bias. Results of the quality assessment are shown in *Table S2* (supporting information).

### Incidence of incidental gallbladder cancer

**Fig. 2:**
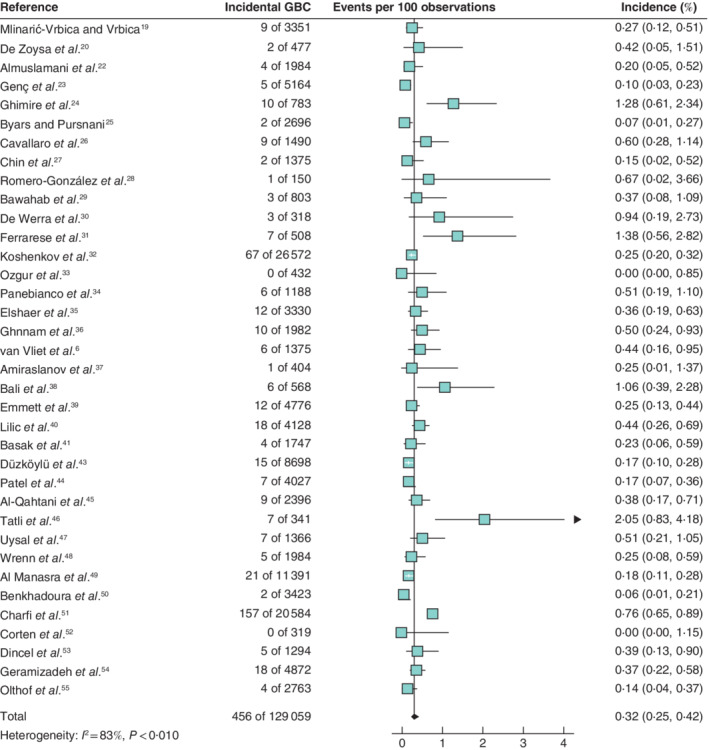
Forest plot of the pooled incidence of incidental gallbladder cancer in low‐incidence countriesA random‐effects model was used for meta‐analysis. Incidence rates are shown with 95 per cent confidence intervals. GBC, gallbladder cancer.

**Fig. 3:**
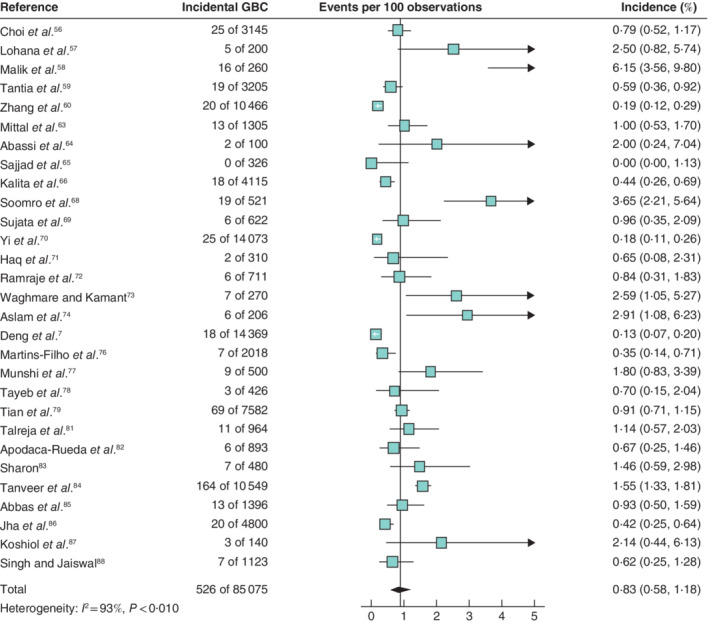
Forest plot of the pooled incidence of incidental gallbladder cancer in high‐incidence countries A random‐effects model was used for meta‐analysis. Incidence rates are shown with 95 per cent confidence intervals. GBC, gallbladder cancer.

Meta‐analysis of 36 studies (129 059 patients) conducted in countries with a low incidence of GBC resulted in a pooled 0·32 (95 per cent c.i. 0·25 to 0·42) per cent incidence of incidental GBC (*Fig*. *2*). Based on 29 studies (88 075 patients) performed in high‐incidence countries, the pooled incidence of incidental GBC was 0·83 (0·58 to 1·18) per cent (*Fig*. *3*).

### Incidence of truly incidental gallbladder cancer

**Fig. 4:**
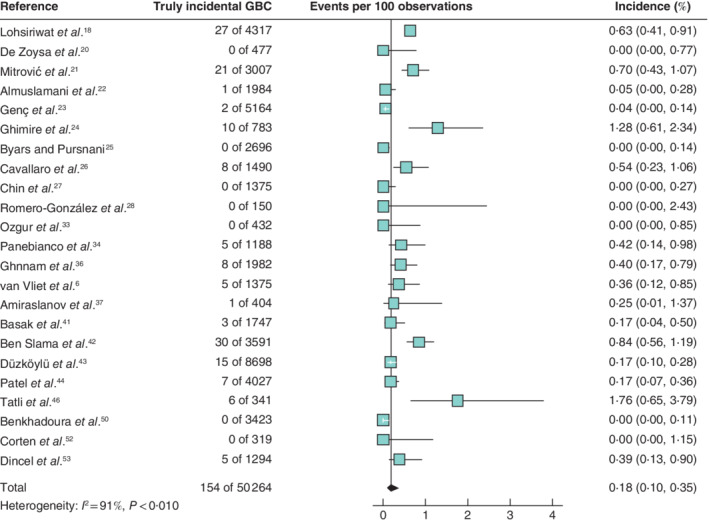
Forest plot of the pooled incidence of truly incidental gallbladder cancer in low‐incidence countries A random‐effects model was used for meta‐analysis. Incidence rates are shown with 95 per cent confidence intervals. GBC, gallbladder cancer.

**Fig. 5:**
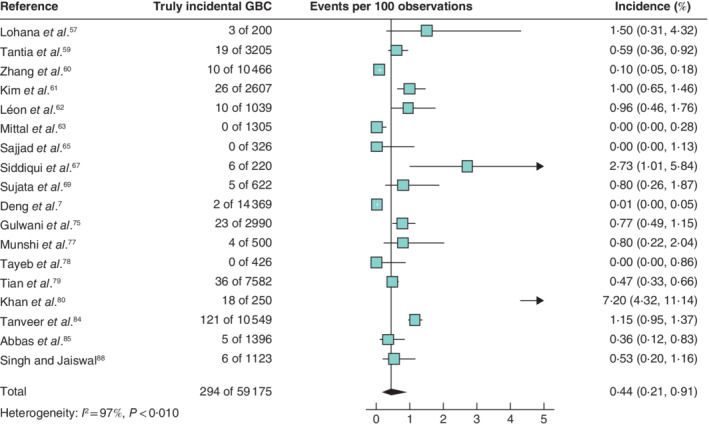
Forest plot of the pooled incidence of truly incidental gallbladder cancer in high‐incidence countries A random‐effects model was used for meta‐analysis. Incidence rates are shown with 95 per cent confidence intervals. GBC, gallbladder cancer.

Based on 23 studies (50 264 patients), the pooled incidence of truly incidental GBC in low‐incidence countries was 0·18 (95 per cent c.i. 0·10 to 0·35) per cent (*Fig*. *4*). Meta‐analysis of 18 studies (59 175 patients) originating from countries with a high incidence of GBC resulted in a 0·44 (0·21 to 0·91) per cent pooled rate of truly incidental GBC (*Fig*. *5*).

Three studies^23^, ^28^, ^52^ (5633 patients) from countries with a low incidence of GBC in which the surgeon systematically inspected the gallbladder mucosa were included in the subgroup analysis. This resulted in an incidence of truly incidental GBC of 0·04 (95 per cent c.i. 0·01 to 0·14) (*Fig. S1*, supporting information). Similar subgroup analysis of six studies^7^, ^60^, ^63^, ^77^, ^78^, ^88^ (28 189 patients) from countries with a high incidence of GBC revealed a pooled incidence of 0·08 (0·02 to 0·39) per cent (*Fig. S2*, supporting information).

### Ability of the surgeon to recognize gallbladder cancer during surgery

Eighteen studies (38 598 patients) performed in countries with a low incidence of GBC reported both the number of GBCs suspected or diagnosed during cholecystectomy (with or without opening the specimen) and the number of GBCs diagnosed during histopathological examination (*Table S3*, supporting information). In these studies, 26·6 (95 per cent c.i. 8·57 to 58·34) per cent (pooled percentage) of incidentally detected cancers were suspected or diagnosed during surgery. In 12 studies (51 743 patients) performed in countries with a high incidence of GBC, the pooled intraoperative sensitivity was 49·5 (95 per cent c.i. 23·7 to 75·5) per cent.

Subgroup analysis of two studies^23^, ^28^ (5314 patients) from countries with a low incidence in which the surgeon systematically examined the gallbladder mucosa showed that surgeons were able to recognize 66·7 (95 per cent c.i. 26·8 to 91·6) per cent (pooled percentage) of incidentally detected cancers. Similar subgroup analysis including six studies^7^, ^60^, ^63^, ^77^, ^78^, ^88^ (28 189 patients) performed in countries with a high incidence of GBC resulted in a pooled intraoperative sensitivity of 76·9 (35·6 to 95·3) per cent.

### Clinical consequences of truly incidental gallbladder cancer

Overall, truly incidental GBC was found in 448 patients among 41 studies including 109 439 patients (*Table S3*, supporting information). Age was provided by 14 studies for 110 patients (24·6 per cent), and sex by 20 studies for 235 patients (52·5 per cent). Median age was 64 (i.q.r. 54–71) years, and 173 patients (73·6 per cent) were women. Nineteen studies^6^, ^7^, ^18^, ^21^, ^22^, ^23^, ^24^, ^26^, ^36^, ^37^, ^41^, ^43^, ^44^, ^53^, ^59^, ^60^, ^67^, ^69^, ^75^ (68 144 patients) including 178 patients with truly incidental GBC reported the clinical consequences of this diagnosis. The consequences for two patients with gallbladder metastases were not described^6^, ^18^. As a result, data for 176 (39·3 per cent) of 448 patients with truly incidental GBC were pooled (*Table* 1). T category was known in 132 (76·3 per cent) of 173 patients with primary GBC: Tis (24, 18·2 per cent), T1a (18, 13·6 per cent), T1b (27, 20·5 per cent), T2 (47, 35·6 per cent) and T3 (16, 12·1 per cent).

**Table 1:** Impact of truly incidental gallbladder cancer on further management

	Total (*n* = 448)	Tis (*n* = 36)	T1a (*n* = 23)	T1b (*n* = 35)	T2 (*n* = 76)	T3 (*n* = 22)	T4 (*n* = 2)	T? (*n* = 249)	Non‐primary GBC (*n* = 5)
**Consequences not described**	272 (60·7)	12 (33)	5 (22)	8 (23)	29 (38)	6 (27)	2 (100)	208 (83·5)	2 (40)
**Consequences described**	176 (39·3)	24 (67)	18 (78)	27 (77)	47 (62)	16 (73)	0 (0)	41 (16·5)	3 (60)
No further treatment	86 (48·9)	17 (71)	7 (39)	9 (33)*	26 (55)†	8 (50)‡		19 (46)	
Periodic surveillance	27 (15·3)	5 (21)§	2 (11)¶	1 (4)#		2 (13)**		17 (42)††	
Referral to oncologist, chemotherapy	15 (8·5)	2 (8)‡‡	1 (6)§§	2 (7)¶¶	2 (4)##	1 (6)***		4 (10)†††	3 (100)‡‡‡
Referral to oncologist, outcome unknown	15 (8·5)		7 (39)	6 (22)	1 (2)	1 (6)			
Secondary surgery, < 1‐year survival	4 (2·3)				3 (6)§§§	1 (6)¶¶¶			
Secondary surgery, unknown survival	16 (9·1)		1 (6)###	5 (19)****	6 (13)††††	3 (19)‡‡‡‡		1 (2)§§§§	
Secondary surgery, ≥ 1‐year survival	13 (7·4)			4 (15)¶¶¶¶	9 (19)####				

Values in parentheses are percentages. Based on data from 41 studies (109 439 patients).

* No further treatment due to refusal by patient (*n* = 1);

† no further treatment due to refusal by patient (*n* = 5), not fit for surgery (*n* = 2), distant metastasis (*n* = 1), unwilling family (*n* = 1);

‡ no further treatment due to poor general condition (*n* = 1);

§ alive at 22, 44, 78, 89 and 125 months (*n* = 5);

¶ alive at 64 and 121 months (*n* = 2);

# alive at 7 months (*n* = 1);

** refused further surgery, died from recurrence at 16 and 19 months (*n* = 2);

†† survival status unknown (*n* = 17);

‡‡ alive at 49 and 62 months (*n* = 2);

§§ survival status unknown (*n* = 1);

¶¶ died at 15 months from unknown cause (*n* = 1), alive at 36 months (*n* = 1);

## alive at 14 months (*n* = 1), survival status unknown (*n* = 1);

*** died from recurrence at 12 months (*n* = 1);

††† survival status unknown (*n* = 4);

‡‡‡ alive at 8 months (*n* = 1), survival status unknown (*n* = 2);

§§§ re‐exploration, no residual disease (died at 12 months from unknown cause, 1; radical surgery, died at 10 and 12 months from unknown cause, 2);

¶¶¶ re‐exploration, residual disease (died at 12 months, 1);

### no details of surgical procedure (*n* = 1);

**** re‐exploration, no residual disease (*n* = 3), revisional surgery with chemotherapy (*n* = 1), revisional surgery (*n* = 1);

†††† re‐exploration, no residual disease (*n* = 4), radical surgery (*n* = 2);

‡‡‡‡ re‐exploration, residual disease (*n* = 2), radical surgery (*n* = 1);

§§§§ radical surgery (*n* = 1);

¶¶¶¶ radical surgery, alive at 32, 36, 38 and 60 months (*n* = 4);

#### radical surgery, alive at 12, 22, 24, 35 and 60 months (*n* = 5), radical surgery, died from recurrence at 57 months (*n* = 1), radical surgery, died at 21, 23 and 30 months from unknown cause (*n* = 3). GBC, gallbladder cancer.

Eighty‐six (48·9 per cent) of the 176 patients did not receive further treatment. Twenty‐seven patients (15·3 per cent) were subjected to periodic surveillance. Fifteen patients (8·5 per cent) were referred to the oncologist and received chemotherapy. For a further 15 patients (8·5 per cent) who were referred to the oncologist, it was not reported whether treatment with chemotherapy was given. Survival data for these patients are provided in *Table* 1. Secondary surgery was performed in 33 patients (18·8 per cent). Survival was unknown in 16 (49 per cent) of these patients. Of the remaining 17 patients, secondary surgery resulted in survival of at least 1 year in 13 patients (77 per cent); the other four patients (24 per cent) had secondary surgery and died within 1 year of the operation.

Details of truly incidental GBC, as reported by studies in which the surgeon systematically examined the gallbladder mucosa, are presented in *Table* 2. In total, 24 patients with truly incidental GBC among nine studies (33 822 patients) were found (*Figs S1* and *S2*, supporting information). T category was known for 18 patients (75 per cent): Tis (8 patients, 44 per cent), T1a (4, 22 per cent), T1b (1, 6 per cent), T2 (3, 17 per cent) and T3 (2, 11 per cent). Clinical consequences were described in three studies^7^, ^23^, ^60^ (29 999 patients), which included 14 (58 per cent) of the 24 patients with truly incidental GBC. T category was T1a or below and T1b or above in eight (57 per cent) and six patients (43 per cent) respectively. Two (14 per cent) of the 14 patients did not have further treatment, and nine (64 per cent) were scheduled for periodic surveillance. Three patients with T2 GBC (21 per cent) underwent radical surgery, which resulted in survival of at least 1 year in all of them.

**Table 2:** Impact of truly incidental gallbladder cancer on further management in studies in which the gallbladder mucosa was examined systematically

	Total (n = 24)	Tis (*n* = 8)	T1a (*n* = 4)	T1b (*n* = 1)	T2 (*n* = 3)	T3 (*n* = 2)	T4 (*n* = 0)	T? (*n* = 6)
**Consequences not described**	10 (42)	2 (25)	2 (50)	0 (0)	0 (0)	0 (0)	0 (0)	6 (100)
**Consequences described**	14 (58)	6 (75)	2 (50)	1 (100)	3 (100)	2 (100)	0 (0)	0 (0)
No further treatment	2 (14)	1 (17)	1 (50)					
Periodic surveillance	9 (64)	5 (83)*	1 (50)†	1 (100)‡		2 (100)§		
Referral to oncologist, chemotherapy								
Referral to oncologist, outcome unknown								
Secondary surgery, < 1‐year survival								
Secondary surgery, unknown survival								
Secondary surgery, ≥ 1‐year survival	3 (21)				3 (100)¶			

Values in parentheses are percentages. Based on data from nine studies (33 822 patients).

* Alive at 22, 44, 78, 89 and 125 months (*n* = 5);

† alive at 121 months (*n* = 1);

‡ alive at 7 months (*n* = 1);

§ refused radical surgery, died from recurrence at 16 and 19 months (*n* = 2);

¶ radical surgery (*n* = 3) (alive at 35 months, 1; died from recurrence at 57 months, 1; died at 21 months from unknown cause, 1).

### Recommendations regarding histopathological examination (routine *versus* selective)

In 20 of the 39 studies conducted in countries with a low incidence of GBC, the authors explicitly mentioned the recommendation regarding histopathological examination (*Table S3*, supporting information). Routine histopathological examination was recommended in nine (45 per cent) of these studies and a selective policy in the remaining 11 (55 per cent). For the 34 high‐incidence countries, such a recommendation was documented in 18 studies. Selective histopathological examination was recommended in only three studies (17 per cent), whereas the remaining 15 (83 per cent) concluded that gallbladder specimens should be assessed routinely by the pathologist.

### Costs

Data from seven studies^23^, ^28^, ^52^, ^60^, ^63^, ^77^, ^78^ (18 330 patients), which reported both the number of specimens with macroscopic abnormalities detected by the surgeon during systematic assessment of the gallbladder and the number of patients with truly incidental GBC, were used to estimate the costs of routine histopathological examination to achieve clinical benefit in one patient. In a hypothetical situation of a selective policy, only 1365 specimens with macroscopic abnormalities (7·4 per cent) would have been sent for further microscopic assessment, whereas 16 965 specimens without macroscopic abnormalities (92·6 per cent) would not have been examined by a pathologist. In 16 of these 16 965 specimens without macroscopic abnormalities (0·1 per cent) truly incidental GBC was diagnosed: Tis category in seven, T1a in three, T1b in one, T2 in three, and T3 in two patients. According to the Dutch guideline, cholecystectomy suffices for Tis and T1a GBC. Therefore, the histopathological diagnosis of GBC might have resulted in additional diagnostic tests and/or treatment in a maximum of six patients. Histopathological costs for a routine policy would have been €1 099 800 (18 330 specimens), compared with €81 900 (1365 specimens) for a selective policy, indicating that the costs of routine histopathological examination for clinical benefit in one patient were at least €169 650.

## Discussion

This meta‐analysis found that the rate of truly incidental GBC following cholecystectomy performed for presumed benign disease was low, especially when the surgeon assessed the mucosa of the removed gallbladder. If not detected during the surgeon's macroscopic assessment of the gallbladder mucosa, GBC was of early stage (T1a category or below) and with no clinical consequence in half of the patients. Based on these results, selective histopathological examination of gallbladder specimens seems safe, at least in non‐endemic regions, and might result in a reduction of more than 90 per cent in the number of gallbladders submitted for examination.

The main argument used for justification of a selective policy is that the diagnosis of incidental GBC is unlikely in gallbladder specimens without macroscopic abnormalities. Several studies^1^, ^2^, ^5^, ^6^, ^25^, ^63^, ^89^ have reported a negative predictive value of 100 per cent when macroscopic abnormalities are absent. Opponents of a selective policy fear that surgeons might not identify these abnormalities, and argue that the macroscopic examination should be performed by a pathologist who is more experienced in recognizing aberrant findings suspicious for GBC. In a prospective study comparing macroscopic gallbladder examination performed by surgeons and pathologists, Corten and colleagues^52^ found strong agreement between both types of assessor (κ = 0·822). Surgeons and pathologists disagreed on only 18 of 319 gallbladder specimens, and no cancer was found in any of these^52^. Similarly, in a prospective study by Romero‐González and co‐workers^28^, surgeons were asked to evaluate the risk of GBC based on clinical risk factors and macroscopic assessment. All three histopathologically confirmed GBCs were recognized by the surgeon. In contrast, the present review showed that GBC was not recognized in at least one‐quarter of patients, despite systematic assessment of the gallbladder mucosa. Surprisingly, two studies^60^, ^88^ reported that T2 and T3 GBCs were missed. Although both studies stated that the gallbladder was opened and examined by the surgeon, it might be assumed that the macroscopic assessment was not performed accurately in these studies, as it is unlikely that these cancers would have no macroscopic abnormalities.

The value of histopathological examination depends mainly on the therapeutic options arising from the diagnosis. No additional treatment is indicated for patients with cancer confined to the epithelium or mucosa of the gallbladder (T1a status or below), provided the resection margin is not involved. For patients with T1b, T2 or T3 GBC, re‐resection to remove any residual disease is recommended, unless this is contraindicated by advanced disease or poor performance status^90^. In the present review, T category was known for 18 of the 24 patients who were diagnosed with truly incidental GBC despite systematic macroscopic examination by the surgeon. Twelve patients were diagnosed with GBC with T1a status or less, meaning that at least 50 per cent (12 of 24) of missed GBC was inconsequential. Histopathological examination revealed GBC of T1b status or above in the remaining six patients, of whom three had additional surgery. Some might state that these patients would have been undertreated if the surgeon had decided to refrain from histopathological examination. However, the clinical impact of secondary surgery on survival has been questioned. Studies^91^, ^92^ have shown that the proportion of patients with residual disease is up to 60 per cent in pT1b/T2 GBC, and exceeds 80 per cent in pT3 GBC. The presence of residual disease is associated with poor survival outcomes, and re‐resection offers only limited survival benefit^93^. In this group of patients, secondary surgery is used as a staging procedure rather than a therapeutic strategy to improve prognosis. Meanwhile, patients without residual disease are exposed to unnecessary surgical risks associated with further resection. Unfortunately, the articles included in this review did not provide data on residual disease or perioperative morbidity. In future studies evaluating the clinical consequences of missed GBC, the balance between potential benefit and harm associated with secondary surgery should be taken into account.

A few other systematic reviews have addressed the question of whether it is necessary to send all gallbladder specimens for histopathological examination. In a review by Jayasundara and de Silva^94^, results of 24 individual studies were summarized narratively. The authors concluded that the level of available evidence was not adequate to recommend selective histopathological examination globally, but that it might be considered in areas with a low incidence of GBC. Similarly, in their review of 21 studies, Jamal *et al*.^17^ suggested that macroscopically normal‐looking gallbladders of patients of European ethnicity under the age of 60 years may be omitted from histopathological examination. In a subanalysis of four studies that performed a detailed examination of the gallbladder mucosa, all 33 GBCs demonstrated macroscopic abnormalities, with 24 of these cancers arising in patients from high‐risk areas^17^. Choi and colleagues^95^ performed a systematic review of 26 studies, including a meta‐analysis, focusing primarily on the incidence and clinical consequences of incidental GBC. The pooled proportion of incidental GBC was 0·7 per cent, and approximately three‐quarters of incidental GBCs were T2 and more advanced cancers. No distinction between GBC detected during or after surgery was made. Consequently, no conclusions on the safety of selective histopathological examination could be drawn.

To comment on the safety of a selective policy, information on the ability of surgeons to identify GBC during surgery is highly relevant. To the authors' knowledge, this is the first meta‐analysis to calculate pooled percentages for both incidental and truly incidental GBC. In addition, subgroup analyses were performed of studies in which surgeons performed a systematic macroscopic assessment of the gallbladder mucosa. The differences between these incidences showed the ability of surgeons to recognize GBC during surgery and the added value of a proper macroscopic assessment of the gallbladder mucosa for identification of GBC. Furthermore, reporting these pooled percentages separately enabled studies to be included as long as they provided the numbers required for at least one intended analysis, resulting in a total of 73 included articles. A final strength of this review is that it provides an estimation of the costs of selective and routine histopathological examination. Although the estimated costs were based on data from only seven studies, and should therefore be interpreted with caution, this review provides insight into the potential cost savings that might result from implementation of selective histopathological examination.

This systematic review is subject to some limitations. Only 11 studies had a prospective design, so the conclusions of the review are based mainly on retrospective data with its inherent shortcomings. A moderate risk of bias was present in most studies, caused mainly by low sample sizes and insufficiently described histopathological methods. The latter limits the robustness of the results presented in this review, as inaccurate histopathological examination and staging of GBC might have biased all reported outcomes. Thirty studies reported on the ability of surgeons to recognize GBC before histopathological examination, but macroscopic examination was performed systematically in only nine, mostly small, studies. The clinical consequences of truly incidental GBC were described for only 176 (39·3 per cent) of all 448 patients. Owing to reporting bias, the actual proportion of diagnoses resulting in a change of postoperative management might be lower. As well as the presence of macroscopic abnormalities, there might be other reasons (such as age, ethnicity, inflamed gallbladder, surgical difficulties) for surgeons to send removed gallbladders for additional assessment by the pathologist. As a result, the number of patients in whom GBC would have been missed with a selective policy might be even lower than reported. Another consequence is that the actual reduction in number of specimens sent for histopathological examination will likely be less than the reported 92·6 per cent. Based on a few single‐centre studies^6^, ^52^, ^55^, selective histopathological examination will probably result in a reduction of 80–90 per cent, but a large prospective cohort of patients would be required to make a valid claim.

When considering a selective policy, one should be aware of regional and ethnic variation in the worldwide distribution of GBC. As expected, different incidences of incidental GBC were observed in the meta‐analyses of studies conducted in low‐ and high‐risk areas. This variation in geographical incidence was reflected in the various authors' recommendations on selective *versus* routine histopathological examination. Whereas more than half of the studies from low‐risk areas suggested a selective policy, the vast majority of authors from high‐risk areas concluded that all gallbladder specimens should be assessed routinely by the pathologist. Owing to the lack of a uniform protocol for pathological examination, considerable variation is observed in the way pathologists conduct the routine assessment of gallbladder specimens^90^, ^96^. In many countries, microscopic examination is not recommended if no abnormalities were found during macroscopic examination. However, for high‐risk populations, international practice guidelines^90^ recommend that the pathologist's assessment should always include microscopic examination of at least three sections and the cystic duct margin, regardless of the presence of macroscopic abnormalities. Previous systematic reviews^17^, ^94^ also concluded that selective histopathological examination can be adopted safely only in regions with a low incidence of GBC. The present review has shown that, even in high‐risk areas, the incidence of truly incidental GBC is only 0·44 per cent, dropping to 0·08 per cent when the gallbladder mucosa is examined systematically by the surgeon. Based on these results, selective histopathological examination might also be safe in countries with a high incidence of GBC, provided the macroscopic assessment is performed carefully. Nonetheless, it should be noted that the incidence of truly incidental GBC in the studies performed in high‐risk areas ranged from 0·0 to 7·2 per cent. As a result, findings of these analyses may not reflect the situation in countries with the highest incidences.

Some might fear the possible medicolegal consequences of a missed incidental GBC. However, the threat of litigation should not justify the overuse of diagnostic tools, especially if the intervention has no benefit for the patient and might potentially even cause harm (such as unnecessary additional resections) and involve substantial costs. Guidelines based on high‐level evidence are required to provide medicolegal protection for surgeons. To gain more evidence on safety and potential cost savings of selective histopathological examination, a large prospective cohort of patients is required. The FANCY study^97^ (NCT03510923) was designed for this reason.

## Supporting information


**Appendix S1:** Supporting information
