# Supplementary material for: Safe, selective histopathological examination of gallbladder specimens: a systematic review
Source: Br J Surg. 2020 Jul 8;107(11):1414–28. doi: 10.1002/bjs.11759 (PMC7540681; doi:10.1002/bjs.11759)

**BJS11759**

**Safe, selective histopathological examination of gallbladder specimens: meta-analysis**

**V. P. Bastiaenen, J. E. Tuijp, S. van Dieren, M. G. Besselink, T. M. van Gulik, L. Koens, P. J. Tanis and W. A. Bemelman**

**Appendix S1** Search details

**PubMed**

(Cholecystitis[MeSH] OR Cholecystitis[tiab] OR Cholecystectomy[MeSH] OR Cholecystectom*[tiab] OR Gallbladder[MeSH] OR Gallbladder*[tiab] OR Gall bladder*[tiab]) AND (Pathology[MeSH] OR Pathology[Subheading] OR Patholog*[tiab] OR Histology[MeSH] OR Histolog*[tiab] OR Histopatholog*[tiab]) AND (Routin*[tiab] OR Selective[tiab] OR Specimen*[tiab]) AND (Carcinoma[MeSH] OR Carcinoma*[tiab] OR Cancer*[tiab])

Filter activated: Publication date from 2009/01/01 to 2019/06/01.

**Embase**

(exp cholecystitis/ or exp cholecystectomy/ or exp gallbladder/ or (cholecystitis or cholecystectom* or gallbladder* or gall bladder*).ti,ab,kw.) and (exp pathology/ or histopathology/ or exp histology/ or (patholog* or histology or histopatholog*).ti,ab,kw.) and ((routin* or selective or specimen*).ti,ab,kw.) and (exp carcinoma/ or (carcinoma* or cancer*).ti,ab,kw.)

Filter activated: Publication date from 2009 to Current.

**Web of Science**

TS=(cholecystitis or cholecystectom* or gallbladder* or gall bladder*) AND TS=(patholog* or histology or histopatholog*) AND TS=(routin* or selective or specimen*) AND TS=(carcinoma* or cancer*)

Filter activated: Publication date from 2009 to Current.

**Cochrane Library**

(cholecystitis or cholecystectom* or gallbladder* or gall bladder*):ti,ab,kw and (patholog* or histology or histopatholog*):ti,ab,kw and (routin* or selective or specimen*):ti,ab,kw and (carcinoma* or cancer*):ti,ab,kw

Filter activated: Publication date from 2009 to Current.

**Table S1 Study characteristics**

|  | **Year of publication** | **Country** | **Study design** | **Study period** | **Number of patients** |
| --- | --- | --- | --- | --- | --- |
| **Studies performed in areas with a low incidence of (incidental) gallbladder cancer (n=39)** | | | | | |
| Lohsiriwat *et al.*^18^ | 2009 | Thailand | Retrospective | 1998-2006 | 4317 |
| Mlinarić-Vrbica *et al.*^19^ | 2009 | Croatia | Retrospective | Not reported | 3351 |
| De Zoysa *et al.*^20^ | 2010 | Sri Lanka | Retrospective | 2007 | 477 |
| Mitrović *et al.*^21^ | 2010 | Bosnia and Herzegovina | Retrospective | Not reported | 3007 |
| Almuslamani *et al.*^22^ | 2011 | Jordan | Retrospective | 2008-2010 | 1984 |
| Genç *et al.*^23^ | 2011 | Turkey | Retrospective | 1999-2010 | 5164 |
| Ghimire *et al.*^24^ | 2011 | Nepal | Retrospective | 1998-2009 | 783 |
| Byars *et al.*^25^ | 2012 | United Kingdom | Retrospective | 2005-2010 | 2696 |
| Cavallaro *et al.*^26^ | 2012 | Italy | Retrospective | 1998-2008 | 1490 |
| Chin *et al.*^27^ | 2012 | Malaysia | Retrospective | 1997-2008 | 1375 |
| Romero-González *et al.*^28^ | 2012 | Mexico | Prospective | 2010-2011 | 150 |
| Bawahab *et al.*^29^ | 2013 | Saudi Arabia | Retrospective | 2010-2012 | 803 |
| De Werra *et al.*^30^ | 2013 | Italy | Retrospective | 2000-2010 | 318 |
| Ferrarese *et al.*^31^ | 2013 | Italy | Retrospective | 2008-2012 | 508 |
| Koshenkov *et al.*^32^ | 2013 | USA | Retrospective | 1996-2011 | 26 572 |
| Ozgur *et al.*^33^ | 2013 | Turkey | Prospective | 2011-2012 | 432 |
| Panebianco *et al.*^34^ | 2013 | Italy | Retrospective | 2003-2011 | 1188 |
| Elshaer *et al.*^35^ | 2014 | United Kingdom | Retrospective | 2004-2012 | 3330 |
| Ghnnam *et al.*^36^ | 2014 | Saudi Arabia | Retrospective | 2007-2012 | 1982 |
| van Vliet *et al.*^6^ | 2014 | The Netherlands | Retrospective | 2007-2011 | 1375 |
| Amiraslanov *et al.*^37^ | 2015 | Azerbaijan | Retrospective | Not reported | 404 |
| Bali *et al.*^38^ | 2015 | Turkey | Retrospective | 2008-2013 | 568 |
| Emmett *et al.*^39^ | 2015 | United Kingdom | Retrospective | 2003-2009 | 4776 |
| Lilic *et al.*^40^ | 2015 | New Zealand | Retrospective | 2003-2013 | 4128 |
| Basak *et al.*^41^ | 2016 | Turkey | Retrospective | 2009-2013 | 1747 |
| Ben Slama *et al.*^42^ | 2016 | Tunisia | Retrospective | 2001-2011 | 3591 |
| Düzköylü *et al.*^43^ | 2016 | Turkey | Retrospective | 2005-2013 | 8698 |
| Patel *et al.*^44^ | 2016 | United Kingdom | Retrospective | 2008-2013 | 4027 |
| Al-Qahtani *et al.*^45^ | 2017 | Saudi Arabia | Retrospective | 2012-2015 | 2396 |
| Tatli *et al.*^46^ | 2017 | Turkey | Retrospective | 2013-2016 | 341 |
| Uysal *et al.*^47^ | 2017 | Turkey | Retrospective | 2011-2017 | 1366 |
| Wrenn *et al.*^48^ | 2017 | USA | Retrospective | 2009-2014 | 1984 |
| Al Manasra *et al.*^49^ | 2018 | Jordan | Retrospective | 2002-2016 | 11 391 |
| Benkhadoura *et al.*^50^ | 2018 | Libya | Retrospective | 2009-2017 | 3423 |
| Charfi *et al.*^51^ | 2018 | Tunisia | Retrospective | 2003-2016 | 20 584 |
| Corten *et al.*^52^ | 2018 | The Netherlands | Prospective | 2009-2011 | 319 |
| Dincel *et al.*^53^ | 2018 | Turkey | Retrospective | 2012-2016 | 1294 |
| Geramizadeh *et al.*^54^ | 2018 | Iran | Retrospective | 2010-2016 | 4872 |
| Olthof *et al.*^55^ | 2018 | The Netherlands | Retrospective | 2011-2017 | 2763 |
| **Studies performed in areas with a high incidence of (incidental) gallbladder cancer (n=34)** | | | | | |
| Choi *et al.*^56^ | 2009 | Korea | Retrospective | 2002-2007 | 3145 |
| Lohana *et al.*^57^ | 2009 | Pakistan | Retrospective | 2006-2008 | 200 |
| Malik *et al.*^58^ | 2009 | Pakistan | Retrospective | 1998-2001 | 260 |
| Tantia *et al.*^59^ | 2009 | India | Retrospective | 2004-2007 | 3205 |
| Zhang *et al.*^60^ | 2009 | China | Retrospective | 1999-2007 | 10 466 |
| Kim *et al.*^61^ | 2010 | Korea | Retrospective | 1997-2008 | 2607 |
| Léon *et al.*^62^ | 2010 | Colombia | Retrospective | 2008-2009 | 1039 |
| Mittal *et al.*^63^ | 2010 | India | Retrospective | 1998-2007 | 1305 |
| Abassi *et al.*^64^ | 2012 | Pakistan | Prospective | 2008 | 100 |
| Sajjad *et al.*^65^ | 2012 | Pakistan | Retrospective | 2006-2010 | 326 |
| Kalita *et al.*^66^ | 2013 | India | Prospective | 2009-2012 | 4115 |
| Siddiqui *et al.*^67^ | 2013 | Pakistan | Prospective | 2010-2012 | 220 |
| Soomro *et al.*^68^ | 2013 | Pakistan | Retrospective | 2007-2008 | 521 |
| Sujata *et al.*^69^ | 2013 | India | Retrospective | 2007-2012 | 622 |
| Yi *et al.*^70^ | 2013 | China | Retrospective | 1992-2009 | 14 073 |
| Haq *et al.*^71^ | 2014 | Pakistan | Retrospective | 2009-2011 | 310 |
| Ramraje *et al.*^72^ | 2014 | India | Retrospective | 2006-2010 | 711 |
| Waghmare *et al.*^73^ | 2014 | India | Retrospective | 2010-2011 | 270 |
| Aslam *et al.*^74^ | 2015 | Pakistan | Prospective | 2013-2014 | 206 |
| Deng *et al.*^7^ | 2015 | China | Retrospective | 2008-2013 | 14 369 |
| Gulwani *et al.*^75^ | 2015 | India | Retrospective | 2001-2013 | 2990 |
| Martins-Filho *et al.*^76^ | 2015 | Brazil | Retrospective | 2007-2010 | 2018 |
| Munshi *et al.*^77^ | 2015 | India | Retrospective | 2011-2012 | 500 |
| Tayeb *et al.*^78^ | 2015 | Pakistan | Prospective | 2009-2012 | 426 |
| Tian *et al.*^79^ | 2015 | China | Retrospective | 2002-2012 | 7582 |
| Khan *et al.*^80^ | 2016 | Pakistan | Prospective | 2014 | 250 |
| Talreja *et al.*^81^ | 2016 | Pakistan | Retrospective | 2005-2015 | 964 |
| Apodaca-Rueda *et al.*^82^ | 2017 | Brazil | Retrospective | 2010-2015 | 893 |
| Sharon *et al.*^83^ | 2017 | India | Retrospective | 2014-2016 | 480 |
| Tanveer *et al.*^84^ | 2017 | Pakistan | Retrospective | 2009-2015 | 10 549 |
| Abbas *et al.*^85^ | 2018 | Pakistan | Retrospective | 2017-2018 | 1396 |
| Jha *et al.*^86^ | 2018 | India | Retrospective | 2014-2016 | 4800 |
| Koshiol *et al.*^87^ | 2018 | Chile | Prospective | 2013-2014 | 140 |
| Singh *et al.*^88^ | 2018 | India | Prospective | 2012-2014 | 1123 |

**Table S2** Quality assessment

|  | | Was the sample frame appropriate to address the target population? | Were study participants sampled in an appropriate way? | Was the sample size adequate? | Were the study subjects and the setting described in detail? | Was the data analysis conducted with sufficient coverage of the identified sample? | Were valid methods used for the identification of the condition? | Was the condition measured in a standard, reliable way for all participants? | Was there appropriate statistical analysis? | Was the response rate adequate, and if not, was the low response rate managed appropriately? | Risk of bias |
| --- | --- | --- | --- | --- | --- | --- | --- | --- | --- | --- | --- |
| **Studies performed in areas with a low incidence of (incidental) gallbladder cancer (n=39)** | | | | | | | | | | | |
| Lohsiriwat *et al.*^18^ | Yes | | Yes | Yes | No | Yes | Yes | Unclear | Yes | Yes | Moderate |
| Mlinarić-Vrbica *et al.*^19^ | Yes | | Yes | Yes | Yes | Yes | Yes | Unclear | Yes | Yes | Low |
| De Zoysa *et al.*^20^ | Yes | | Yes | No | No | Yes | Yes | Unclear | Yes | Yes | Moderate |
| Mitrović *et al.*^21^ | Yes | | Yes | Yes | No | Yes | Yes | Unclear | Yes | Yes | Moderate |
| Almuslamani *et al.*^22^ | Yes | | Yes | No | No | Yes | Yes | Unclear | Yes | Yes | Moderate |
| Genç *et al.*^23^ | Yes | | Yes | Yes | Yes | Yes | Yes | Unclear | Yes | Yes | Low |
| Ghimire *et al.*^24^ | Yes | | Yes | No | Yes | Yes | Yes | Unclear | Yes | Yes | Moderate |
| Byars *et al.*^25^ | Yes | | Yes | No | Yes | Yes | Yes | Unclear | Yes | Yes | Moderate |
| Cavallaro *et al.*^26^ | Yes | | Yes | No | No | Yes | Yes | Unclear | Yes | Yes | Moderate |
| Chin *et al.*^27^ | Yes | | Yes | No | Yes | Yes | Yes | Unclear | Yes | Yes | Moderate |
| Romero-González *et al.*^28^ | Yes | | Yes | No | Yes | Yes | Yes | Yes | Yes | Yes | Low |
| Bawahab *et al.*^29^ | Yes | | Yes | No | Yes | Yes | Yes | Unclear | Yes | Yes | Moderate |
| De Werra *et al.*^30^ | Yes | | Yes | No | Yes | Yes | Yes | Unclear | Yes | Yes | Moderate |
| Ferrarese *et al.*^31^ | Yes | | Yes | No | No | Yes | Yes | Unclear | Yes | Yes | Moderate |
| Koshenkov *et al.*^32^ | Yes | | Yes | Yes | No | Yes | Yes | Unclear | Yes | Yes | Moderate |
| Ozgur *et al.*^33^ | Yes | | Unclear | No | Yes | Unclear | Yes | Yes | Yes | Yes | Moderate |
| Panebianco *et al.*^34^ | Yes | | Yes | No | Yes | Yes | Yes | Unclear | Yes | Yes | Moderate |
| Elshaer *et al.*^35^ | Yes | | Yes | Yes | No | Yes | Yes | Unclear | Yes | Yes | Moderate |
| Ghnnam *et al.*^36^ | Yes | | Yes | No | No | Yes | Yes | Unclear | Yes | Yes | Moderate |
| van Vliet *et al.*^6^ | Yes | | Yes | No | No | Yes | Yes | Unclear | Yes | Yes | Moderate |
| Amiraslanov *et al.*^37^ | Unclear | | Unclear | No | No | Unclear | Yes | Yes | Yes | Yes | High |
| Bali *et al.*^38^ | Yes | | Yes | No | Yes | Yes | Yes | Yes | Yes | Yes | Low |
| Emmett *et al.*^39^ | Yes | | Yes | Yes | Yes | Yes | Yes | Unclear | Yes | Yes | Low |
| Lilic *et al.*^40^ | Yes | | Yes | Yes | No | Yes | Yes | Unclear | Yes | Yes | Moderate |
| Basak *et al.*^41^ | Yes | | Yes | No | Yes | Yes | Yes | Unclear | Yes | Yes | Moderate |
| Ben Slama *et al.*^42^ | Yes | | Yes | Yes | No | Yes | Yes | Yes | Yes | Yes | Low |
| Düzköylü *et al.*^43^ | Yes | | Yes | Yes | No | Yes | Yes | Unclear | Yes | Yes | Moderate |
| Patel *et al.*^44^ | Yes | | Yes | Yes | No | Yes | Yes | No | Yes | Yes | Moderate |
| Al-Qahtani *et al.*^45^ | Yes | | Yes | No | Yes | Yes | Yes | Unclear | Yes | Yes | Moderate |
| Tatli *et al.*^46^ | Yes | | Yes | No | Yes | Yes | Yes | Unclear | Yes | Yes | Moderate |
| Uysal *et al.*^47^ | Yes | | Yes | No | Yes | Yes | Yes | Unclear | Yes | Yes | Moderate |
| Wrenn *et al.*^48^ | Yes | | Yes | No | Yes | Yes | Yes | Yes | Yes | Yes | Low |
| Al Manasra *et al.*^49^ | Yes | | Yes | Yes | No | Yes | Yes | No | No | Yes | Moderate |
| Benkhadoura *et al.*^50^ | Yes | | Yes | Yes | Yes | Yes | Yes | No | Yes | Yes | Low |
| Charfi *et al.*^51^ | Yes | | Yes | Yes | Yes | Yes | Yes | Yes | Yes | Yes | Low |
| Corten *et al.*^52^ | Yes | | Yes | No | Yes | Yes | Yes | Yes | Yes | Yes | Low |
| Dincel *et al.*^53^ | Yes | | Yes | No | Yes | Yes | Yes | No | Yes | Yes | Moderate |
| Geramizadeh *et al.*^54^ | Yes | | Yes | Yes | Yes | Yes | Yes | No | Yes | Yes | Low |
| Olthof *et al.*^55^ | Yes | | Yes | No | Yes | Yes | Yes | Yes | Yes | Yes | Low |
| **Studies performed in areas with a high incidence of (incidental) gallbladder cancer (n=34)** | | | | | | | | | | | |
| Choi *et al.*^56^ | Yes | | Yes | Yes | No | Yes | Yes | Unclear | Yes | Yes | Moderate |
| Lohana *et al.*^57^ | Yes | | Unclear | No | No | Unclear | Yes | Unclear | Yes | Yes | High |
| Malik *et al.*^58^ | Yes | | Yes | No | No | Yes | Yes | Unclear | Yes | Yes | Moderate |
| Tantia *et al.*^59^ | Yes | | Yes | Yes | No | Yes | Yes | Unclear | Yes | Yes | Moderate |
| Zhang *et al.*^60^ | Yes | | Yes | Yes | No | Yes | Yes | Unclear | Yes | Yes | Moderate |
| Kim *et al.*^61^ | Yes | | Yes | No | No | Yes | Yes | Unclear | Yes | Yes | Moderate |
| Léon *et al.*^62^ | Yes | | Yes | No | Yes | Yes | Yes | Unclear | Yes | Yes | Moderate |
| Mittal *et al.*^63^ | Yes | | Yes | No | Yes | Yes | Yes | Unclear | Yes | Yes | Moderate |
| Abassi *et al.*^64^ | Yes | | Yes | No | Yes | Yes | Yes | Unclear | Yes | Yes | Moderate |
| Sajjad *et al.*^65^ | Yes | | Yes | No | Yes | Yes | Yes | Yes | Yes | Yes | Low |
| Kalita *et al.*^66^ | Unclear | | Unclear | Yes | No | Unclear | Yes | Yes | Yes | Yes | Moderate |
| Siddiqui *et al.*^67^ | Yes | | Yes | No | Yes | Yes | Yes | Unclear | Yes | Yes | Moderate |
| Soomro *et al.*^68^ | Yes | | Yes | No | Yes | Yes | Yes | Unclear | Yes | Yes | Moderate |
| Sujata *et al.*^69^ | Yes | | Yes | No | No | Yes | Yes | Yes | Yes | Yes | Moderate |
| Yi *et al.*^70^ | Unclear | | Yes | Yes | No | Yes | Yes | Unclear | Yes | Yes | Moderate |
| Haq *et al.*^71^ | No | | Yes | No | Yes | Yes | Yes | Unclear | Yes | Yes | Moderate |
| Ramraje *et al.*^72^ | Yes | | Unclear | No | No | Unclear | Yes | Unclear | Yes | Yes | High |
| Waghmare *et al.*^73^ | Yes | | Yes | No | No | Yes | Yes | Unclear | Yes | Yes | Moderate |
| Aslam *et al.*^74^ | Yes | | Yes | No | Yes | Yes | Yes | Unclear | Yes | Yes | Moderate |
| Deng *et al.*^7^ | Yes | | Yes | Yes | Yes | Yes | Yes | Unclear | Yes | Yes | Low |
| Gulwani *et al.*^75^ | Yes | | Yes | Yes | No | Yes | Yes | Yes | Yes | Yes | Low |
| Martins-Filho *et al.*^76^ | Yes | | Yes | No | No | Yes | Yes | Unclear | Yes | Yes | Moderate |
| Munshi *et al.*^77^ | Yes | | Yes | No | Yes | Yes | Yes | Unclear | Yes | Yes | Moderate |
| Tayeb *et al.*^78^ | Yes | | Yes | No | Yes | Yes | Yes | Yes | Yes | Yes | Low |
| Tian *et al.*^79^ | Yes | | Yes | Yes | No | Yes | Yes | Unclear | Yes | Yes | Moderate |
| Khan *et al.*^80^ | Yes | | Yes | No | Yes | Yes | Yes | Unclear | Yes | Yes | Moderate |
| Talreja *et al.*^81^ | Yes | | Yes | No | Yes | Yes | Yes | Unclear | Yes | Yes | Moderate |
| Apodaca-Rueda *et al.*^82^ | Yes | | Yes | No | Yes | Yes | Yes | Unclear | Yes | Yes | Moderate |
| Sharon *et al.*^83^ | Yes | | Yes | No | No | Yes | Yes | Unclear | Yes | Yes | Moderate |
| Tanveer *et al.*^84^ | Yes | | Yes | Yes | Yes | Yes | Yes | Yes | Yes | Yes | Low |
| Abbas *et al.*^85^ | Yes | | Unclear | No | No | Unclear | Yes | Unclear | Yes | Yes | High |
| Jha *et al.*^86^ | Yes | | Yes | Yes | Yes | Yes | Yes | Unclear | Yes | Yes | Low |
| Koshiol *et al.*^87^ | No | | Yes | No | Yes | Yes | Yes | Yes | Yes | Yes | Moderate |
| Singh *et al.*^88^ | Yes | | Yes | No | Yes | Yes | Yes | Unclear | Yes | Yes | Moderate |

**Table S3** Diagnosis of incidental gallbladder cancer and recommendation for histopathological examination

|  | **Number of patients** | **Preoperatively unsuspected GBC, No. (%)** | **Primary GBC** | | | | | | | **Non-primary GBC** | | **Suspicion/Diagnosis** | | **Recommendation** |
| --- | --- | --- | --- | --- | --- | --- | --- | --- | --- | --- | --- | --- | --- | --- |
|  |  |  | **Tis** | **T1a** | **T1b** | **T2** | **T3** | **T4** | **T?** | **Lymphoid malignancies** | **Gallbladder metastases** | **Intraoperative**  **No. (%)** | **Postoperative**  **No (%)** |  |
| **Studies performed in areas with a low incidence of (incidental) gallbladder cancer (n=39)** | | | | | | | | | | | | | | |
| Lohsiriwat *et al.*^18^ | 4317 | 27 (0·6%)* | - | 11 | | 11 | 2 | - | - | - | 3 | Excluded | 27 | Routine |
| Mlinarić-Vrbica *et al.*^19^ | 3351 | 9 (0·3%) | - | - | - | - | - | - | 9 | - | - | 9 intra- or postoperative | | Not reported |
| De Zoysa *et al.*^20^ | 477 | 2 (0·4%) | - | - | - | - | - | - | 2 | - | - | 2 | 0 | Not reported |
| Mitrović *et al.*^21^ | 3007 | 21 (0·7%)* | 4 | 3 | | 12 | | - | 2 | - | - | Excluded | 21 | Not reported |
| Almuslamani *et al.*^22^ | 1984 | 4 (0·2%) | 1 | - | - | 1 | 2 | - | - | - | - | 3 | 1 | Selective |
| Genç *et al.*^23^ | 5164 | 5 (0·1%) | 1 | - | 1 | 1 | 2 | - | - | - | - | 3 | 2 | Not reported |
| Ghimire *et al.*^24^ | 783 | 10 (1·3%) | - | 3 | 5 | 2 | - | - | - | - | - | 0 | 10 | Routine |
| Byars *et al.*^25^ | 2696 | 2 (0·1%) | - | - | - | - | - | - | 2 | - | - | 2 | 0 | Selective |
| Cavallaro *et al.*^26^ | 1490 | 9 (0·6%) | 1 | 1 | 3 | 4 | - | - | - | - | - | 1 | 8 | Not reported |
| Chin *et al.*^27^ | 1375 | 2 (0·1%) | - | 2 | | - | - | - | - | - | - | 2 | 0 | Selective |
| Romero-González *et al.*^28^ | 150 | 1 (0·7%) | - | - | - | - | 1 | - | - | - | - | 1 | 0 | Selective |
| Bawahab *et al.*^29^ | 803 | 3 (0·4%) | - | - | - | 1 | 2 | - | - | - | - | 3 intra- or postoperative | | Selective |
| De Werra *et al.*^30^ | 318 | 3 (0·9%) | 1 | - | 1 | - | 1 | - | - | - | - | 3 intra- or postoperative | | Not reported |
| Ferrarese *et al.*^31^ | 508 | 7 (1·4%) | - | - | 1 | 4 | 2 | - | - | - | - | 7 intra- or postoperative | | Not reported |
| Koshenkov *et al.*^32^ | 26 572 | 67 (0·3%) | 6 | 10 | | 31 | 14 | - | 6 | - | - | 67 intra- or postoperative | | Not reported |
| Ozgur *et al.*^33^ | 432 | 0 (0·0%) | - | - | - | - | - | - | - | - | - | 0 | 0 | Not reported |
| Panebianco *et al.*^34^ | 1188 | 6 (0·5%) | - | 1 | | 2 | 3 | - | - | - | - | 1 | 5 | Not reported |
| Elshaer *et al.*^35^ | 3330 | 12 (0·4%) | 1 | - | 1 | - | 6 | 1 | - | 2^†^ | 1 | 12 intra- or postoperative | | Not reported |
| Ghnnam *et al.*^36^ | 1982 | 10 (0·5%) | - | 4 | 2 | 2 | 2 | - | - | - | - | 2 | 8 | Routine |
| van Vliet *et al.*^6^ | 1375 | 6 (0·4%) | - | - | - | 3 | 2 | - | - | - | 1 | 1 | 5 | Selective |
| Amiraslanov *et al.*^37^ | 404 | 1 (0·2%) | - | - | 1 | - | - | - | - | - | - | 0 | 1 | Routine |
| Bali *et al.*^38^ | 568 | 6 (1·1%) | - | 2 | 1 | 3 | - | - | - | - | - | 6 intra- or postoperative | | Not reported |
| Emmett *et al.*^39^ | 4776 | 12 (0·3%) | - | - | - | - | - | - | 12 | - | - | 12 intra- or postoperative | | Selective |
| Lilic *et al.*^40^ | 4128 | 18 (0·4%) | - | - | - | - | - | - | 18 | - | - | 18 intra- or postoperative | | Not reported |
| Basak *et al.*^41^ | 1747 | 4 (0·2%) | - | - | - | 3 | 1 | - | - | - | - | 1 | 3 | Routine |
| Ben Slama *et al.*^42^ | 3591 | 30 (0·8%)* | 7 | 0 | 5 | 12 | 4 | 2 | - | - | - | Excluded | 30 | Routine |
| Düzköylü *et al.*^43^ | 8698 | 15 (0·2%) | 3 | 1 | 2 | 5 | 3 | - | - | 1^‡^ | - | 0 | 15 | Not reported |
| Patel *et al.*^44^ | 4027 | 7 (0·2%) | 1 | 1 | 1 | 2 | 2 | - | - | - | - | 0 | 7 | Routine |
| Al-Qahtani *et al.*^45^ | 2396 | 9 (0·4%) | - | - | 5 | 3 | 1 | - | - | - | - | 9 intra- or postoperative | | Selective |
| Tatli *et al.*^46^ | 341 | 7 (2·1%) | - | - | 3 | 4 | - | - | - | - | - | 1 | 6 | Not reported |
| Uysal *et al.*^47^ | 1366 | 7 (0·5%) | 2 | 1 | | 2 | 1 | 1 | - | - | - | 7 intra- or postoperative | | Routine |
| Wrenn *et al.*^48^ | 1984 | 5 (0·3%) | - | - | - | 2 | 3 | - | - | - | - | 5 intra- or postoperative | | Not reported |
| Al Manasra *et al.*^49^ | 11 391 | 21 (0·2%) | - | - | - | - | - | - | 21 | - | - | 21 intra- or postoperative | | Not reported |
| Benkhadoura *et al.*^50^ | 3423 | 2 (0·1%) | - | - | - | 2 | - | - | - | - | - | 2 | 0 | Selective |
| Charfi *et al.*^51^ | 20 584 | 157 (0·8%) | - | 18 | | 68 | 36 | - | 33 | 2^$^ | - | 157 intra- or postoperative | | Not reported |
| Corten *et al.*^52^ | 319 | 0 (0·0%) | - | - | - | - | - | - | - | - | - | 0 | 0 | Selective |
| Dincel *et al.*^53^ | 1294 | 5 (0·4%) | - | - | - | 4 | 1 | - | - | - | - | 0 | 5 | Routine |
| Geramizadeh *et al.*^54^ | 4872 | 18 (0·4%) | - | 10 | | 8 | - | - | - | - | - | 18 intra- or postoperative | | Not reported |
| Olthof *et al.*^55^ | 2763 | 4 (0·1%) | - | - | - | 3 | - | - | - | - | 1 | 4 intra- or postoperative | | Selective |
| **Studies performed in areas with a high incidence of (incidental) gallbladder cancer (n=34)** | | | | | | | | | | | | | | |
| Choi *et al.*^56^ | 3145 | 25 (0·8%) | - | - | - | - | - | - | 25 | - | - | 25 intra- or postoperative | | Not reported |
| Lohana *et al.*^57^ | 200 | 5 (2·5%) | - | - | - | - | - | - | 5 | - | - | 2 | 3 | Routine |
| Malik *et al.*^58^ | 260 | 16 (6·2%) | - | 11 | | 3 | 2 | - | - | - | - | 16 intra- or postoperative | | Not reported |
| Tantia *et al.*^59^ | 3205 | 19 (0·6%) | 8 | 4 | 4 | 3 | - | - | - | - | - | 0 | 19 | Not reported |
| Zhang *et al.*^60^ | 10 466 | 20 (0·2%) | 4 | 2 | 2 | 6 | 4 | 2 | - | - | - | 10 | 10 | Not reported |
| Kim *et al.*^61^ | 2607 | 26 (1·0%)* | 1 | 3 | 3 | 17 | 2 | - | - | - | - | Excluded | 26 | Not reported |
| Léon *et al.*^62^ | 1039 | 10 (1·0%)* | 2 | - | - | - | - | - | 8 | - | - | Not reported^¶^ | 10 | Not reported |
| Mittal *et al.*^63^ | 1305 | 13 (1·0%) | 1 | - | - | 9 | 3 | - | - | - | - | 13 | 0 | Selective |
| Abassi *et al.*^64^ | 100 | 2 (2·0%) | - | - | - | - | - | - | 2 | - | - | 2 intra- or postoperative | | Routine |
| Sajjad *et al.*^65^ | 326 | 0 (0·0%) | - | - | - | - | - | - | - | - | - | 0 | 0 | Routine |
| Kalita *et al.*^66^ | 4115 | 18 (0·4%) | 1 | - | 7 | 10 | - | - | - | - | - | 18 intra- or postoperative | | Routine |
| Siddiqui *et al.*^67^ | 220 | 6 (2·7%)* | - | - | 3 | 2 | 1 | - | - | - | - | Excluded | 6 | Routine |
| Soomro *et al.*^68^ | 521 | 19 (3·6%) | - | - | - | - | - | - | 19 | - | - | 19 intra- or postoperative | | Routine |
| Sujata *et al.*^69^ | 622 | 6 (1·0%) | - | 3 | 1 | 1 | 1 | - | - | - | - | 1 | 5 | Routine |
| Yi *et al.*^70^ | 14 073 | 25 (0·2%) | - | - | - |  | - | - | 25 | - | - | 25 intra- or postoperative | | Not reported |
| Haq *et al.*^71^ | 310 | 2 (0·6%) | - | - | - | - | - | - | 2 | - | - | 2 intra- or postoperative | | Not reported |
| Ramraje *et al.*^72^ | 711 | 6 (0·8%) | - | - | - | - | - | - | 6 | - | - | 6 intra- or postoperative | | Routine |
| Waghmare *et al.*^73^ | 270 | 7 (2·6%) | - | 1 | - | 6 | - | - | - | - | - | 7 intra- or postoperative | | Not reported |
| Aslam *et al.*^74^ | 206 | 6 (2·9%) | - | 5 | 1 | - | - | - | - | - | - | 6 intra- or postoperative | | Routine |
| Deng *et al.*^7^ | 14 369 | 18 0·1%) | 4 | 1 | 2 | - | 11 | - | - | - | - | 16 | 2 | Selective |
| Gulwani *et al.*^75^ | 2990 | 23 (0·8%)* | - | - | 5 | 14 | 4 | - | - | - | - | Excluded | 23 | Not reported |
| Martins-Filho *et al.*^76^ | 2018 | 7 (0·3%) | - | 1 | 1 | 5 | - | - | - | - | - | 7 intra- or postoperative | | Not reported |
| Munshi *et al.*^77^ | 500 | 9 (1·8%) | 2 | 2 | 3 | 2 | - | - | - | - | - | 5 | 4 | Routine |
| Tayeb *et al.*^78^ | 426 | 3 (0·7%) | - | 1 | | 1 | 1 | - | - |  | - | 3 | 0 | Selective |
| Tian *et al.*^79^ | 7582 | 69 (0·9%) | - | 22 | 16 | 13 | 18 | - | - | - | - | 33 | 36 | Not reported |
| Khan *et al.*^80^ | 250 | 18 (7·2%)* | - | - | - | - | - | - | 18 | - | - | Excluded | 18 | Routine |
| Talreja *et al.*^81^ | 964 | 11 (1·1%) | 1 | 2 | 2 | 6 | - | - | - | - | - | 11 intra- or postoperative | | Not reported |
| Apodaca-Rueda *et al.*^82^ | 893 | 6 (0·7%) | 5 | - | - | - | - | - | - | 1^#^ | - | 6 intra- or postoperative | | Not reported |
| Sharon *et al.*^83^ | 480 | 7 (1·5%) | - | - | - | - | - | - | 7 | - | - | 7 intra- or postoperative | | Not reported |
| Tanveer *et al.*^84^ | 10 549 | 164 (1·6%) | - | - | - | - | - | - | 164 | - | - | 43 | 121 | Routine |
| Abbas *et al.*^85^ | 1396 | 13 (0·9%) | - | - | - | - | - | - | 13 | - | - | 8 | 5 | Routine |
| Jha *et al.*^86^ | 4800 | 20 (0·4%) | - | 3 | 15 | 2 | - | - | - | - | - | 20 intra- or postoperative | | Routine |
| Koshiol *et al.*^87^ | 140 | 3 (2·1%) | - | - | - | 3 | - | - | - | - | - | 3 intra- or postoperative | | Not reported |
| Singh *et al.*^88^ | 1123 | 7 (0·6%) | - | 1 | 3 | 3 | - | - | - | - | - | 1 | 6 | Routine |
| GBC, gallbladder cancer | | | | | | | | | | | | | | |
| * The actual number of patients with pre-operatively unsuspected might be higher since intraoperative suspected or diagnosed GBC were excluded. | | | | | | | | | | | | | | |
| † Non-Hodgkin lymphoma (n=1), chronic lymphocytic leukemia (n=1) | | | | | | | | | | | | | | |
| ‡ Mucosa-associated lymphoid tissue (MALT) lymphoma (n=1) | | | | | | | | | | | | | | |
| $ Lymphoma, not further specified (n=2) | | | | | | | | | | | | | | |
| ¶ A total of four gallbladder cancers were pre- or intraoperatively detected (distinction between pre- and intraoperative was not made). | | | | | | | | | | | | | | |
| # Non-Hodgkin lymphoma (n=1) | | | | | | | | | | | | | | |

**Fig. S1** Pooled incidence of truly incidental gallbladder cancer in low-incidence countries (subgroup)


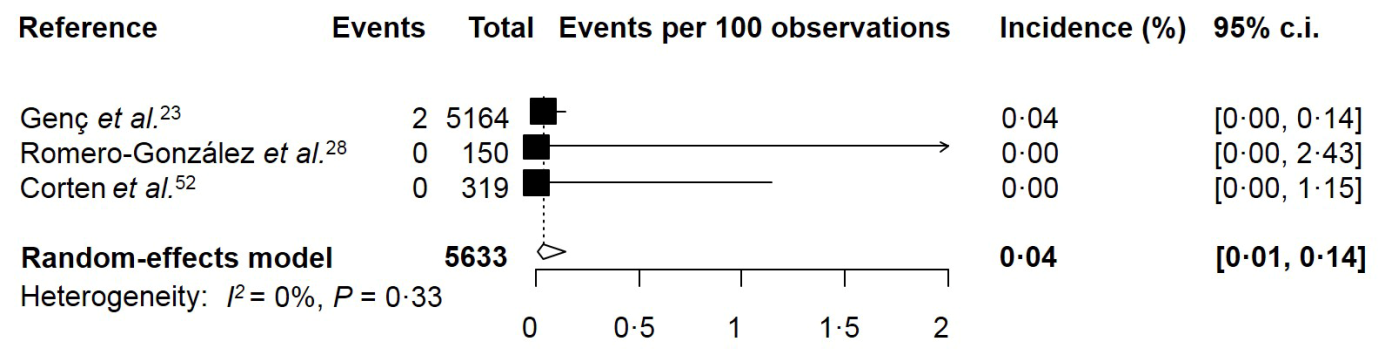


**Fig. S2** Pooled incidence of truly incidental gallbladder cancer in high-incidence countries (subgroup)


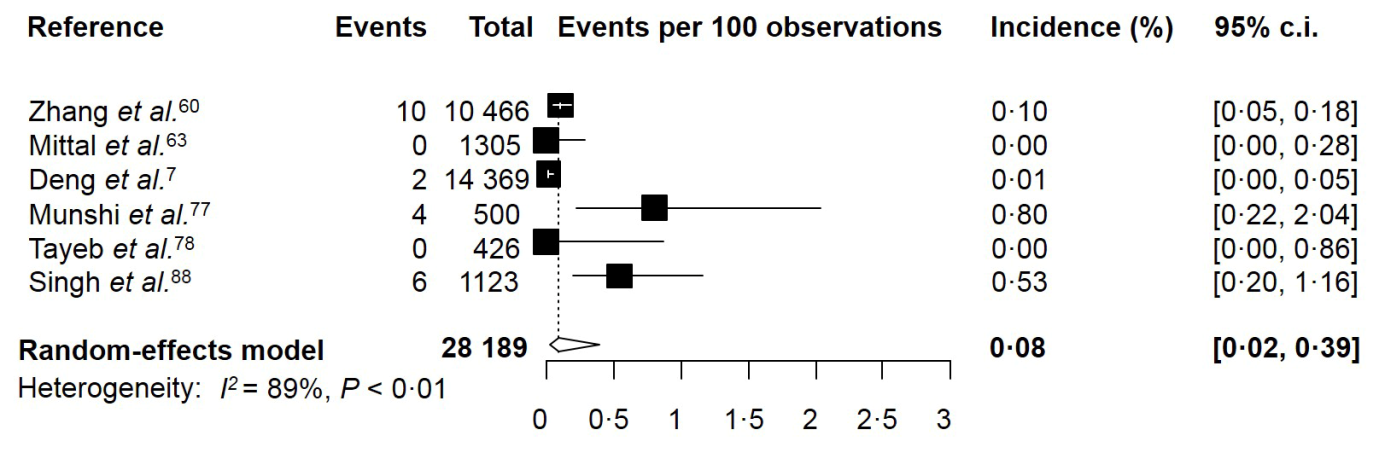

Supplement: Supplementary file 1 — Appendix S1: Supporting information [file BJS-107-1414-s001.docx]
